# Insured but unequal: who really utilizes voluntary health insurance in Israel? Evidence from two consecutive cross-sectional studies

**DOI:** 10.1186/s13584-026-00763-2

**Published:** 2026-05-29

**Authors:** Ruth Waitzberg, Rina Maoz-Breuer, Ella Katz, Nathan Shuftan

**Affiliations:** 1 The Smokler Center for Health Policy Research, Myers-JDC-Brookdale Institute, Jerusalem, Israel; 2 Department of Health Care Management, Faculty of Economics and Management, Technische Universität Berlin, Straße des 17. Juni 135, Berlin, 10623 Germany

**Keywords:** Voluntary health insurance, Access, Health financing, Israel, Utilization

## Abstract

**Background:**

Financial pressures on health systems have increased in recent years. Voluntary health insurance (VHI) could, in principle, fill in the gaps in public coverage and funding. However, there is little evidence on realized access to VHI-funded care – who uses it and for what services, especially in countries with high VHI ownership, such as Israel. Our study assesses gaps in VHI uptake and utilization across the Israeli population.

**Methods:**

Two consecutive cross-sectional data were collected through two national surveys among the adult population (aged 22+) conducted in 2012 and again in 2022, with respective response rates of 61% (N= 2,330) and 52% (*N* = 2,536). Bivariate analyses (ꭓ^2^) estimated the differences between population groups in the rates of VHI ownership and utilization of services at least once during the two years preceding the survey, while multivariable logistic regressions estimated the corresponding odds ratios.

**Findings:**

VHI ownership rates remained high (around 83%) in both surveys but varied across subgroups. Arabs, residents of peripheral areas, and those in the lowest income quintile had lower ownership rates and lower likelihood of owning it. Overall, VHI self-reported utilization stood at 66% in 2022, lower than 76% in 2012. Visits to specialists were the only service category with increased utilization (from 20% to 28%). In 2022, VHI owners with lower incomes, lower education, and those residing in peripheral areas were also less likely to report utilizing VHI. Yet, Arabs and ultra-Orthodox Jews were more likely to report utilizing VHI. Those reporting poor health were also more likely to report utilizing VHI.

**Discussion and conclusions:**

Our study examines VHI ownership trends and gaps in utilization of VHI-funded services in Israeli populations. The regressive nature of VHI premiums and disproportionately higher utilization by owners with higher socioeconomic status highlights the limitations of VHI as a sustainable and equitable financing policy tool for healthcare to policymakers. This unequal coverage demonstrates that VHI is not a full substitute for public funds, potentially indicating access barriers, while showing that fewer insured are benefiting from coverage. Its role should be clearly defined to ensure it complements public healthcare coverage.

**Supplementary Information:**

The online version contains supplementary material available at 10.1186/s13584-026-00763-2.

## Introduction

Health systems’ financial pressures have been exacerbated in recent years by shocks such as rising and changing disease burdens, pandemics [1], armed conflicts, natural disasters and economic crises. Since 2021, several high-income countries (HICs) have faced economic downturns and rising inflation, putting additional strain on health systems’ capacities and fiscal space. Lessons from Europe’s economic shock in 2008 and the COVID-19 pandemic highlight how health systems were weakened due to reduced public revenues along with increased need for publicly financed healthcare [2], or by increased public debt due to borrowing [3]. The current challenges of sticky inflation and economic stagnation in Europe and other HICs [4] are likely to further endanger countries’ public spending levels on healthcare and limit coverage [5]. In parallel, to fill the gaps of public coverage and financing, private spending in the form of out-of-pocket (OOP) and voluntary health insurance (VHI) are likely to rise [6].

In several HICs, VHI is already widespread and covers more than half of the population, including Belgium, France, the Netherlands, Slovenia (until 2024), South Korea, Canada, Luxembourg and Israel [7]. In France (and previously Slovenia), the government even subsidizes VHI to the poor, as a measure to mitigate inequities in access to care [8]. In Israel, Canada, and Slovenia, VHI has played an important role and financed 10% or more of current health expenditures (CHE) in 2023, whereas the average across the EU27 countries was just 4.5% [9]. In OECD countries, in the period just before the COVID-19 pandemic (2015–2019), annual average growth rates (real terms) in per capita VHI expenditures (5.6%) were much higher than those of government-financed schemes (1.3%), compulsory contributory schemes (3.5%) or even OOP payments (1.8%) [7].

In this context, VHI as prepaid funds could, in principle and if set as a priority, substitute public coverage and funding. Yet, little is known about the extent that VHI can perform all the roles of public insurance, including financial protection and equity. While it is well known who owns VHI [10, 11], there is little evidence on who utilizes it. In general, there is a lack of recent research on equity in healthcare financing in HICs [12]; most studies use progressivity indices (e.g. Kakwani) to assess the equity of healthcare financing mechanisms in relation to people’s income [13–15]. However, these indices have limitations, as they fail to account for access to healthcare services. They do not reveal how healthcare service utilization is distributed across income groups and should be complemented by data on access to care according to income levels [12]. In addition, there is little evidence of the horizontal equity in access to services financed by VHI, particularly for the poor and other vulnerable populations who hold such insurance.

Access is defined by WHO as “the opportunity or ability to use comprehensive, appropriate, timely, quality health services when they are needed” [16, 17]. Access to care is the result of three main dimensions, namely affordability, availability and acceptability[17, 18]. In this first exploratory study, we assess utilization of VHI services as an indicator of realized access to health services funded by VHI. Our work fills a gap in the literature by providing new insights on disparities in utilization of VHI in a HIC with high levels of VHI ownership (Israel). We assess VHI uptake and utilization to pay for services in two time points (2012 and 2022), across different population groups.

### Israel as a case study

Israel’s broad VHI market is unique and can serve as a useful case study. VHI plays a significant role in the health system, accounting for 12% of current spending on health and 32% of private spending on health in 2022 [19]. VHI expands the public coverage provided by the Israeli National Health Insurance (NHI) system, by extending coverage to services that are otherwise only partially covered or not at all. VHI also duplicates NHI coverage by offering a wider choice, greater availability and improved access to covered services. VHI in Israel does not cover user charges for NHI-covered services [20]. The NHI allows HPs to charge user charges for certain services such as prescription outpatient medicines or visits to specialists. They are regulated by the Ministry of Health (MoH), but health plans (HP) can define the level of user charges. To protect vulnerable people from financial hardship, discounts, exemptions and caps are set, for example for people with certain chronic conditions, older adults or children receiving income support allowances [21–23].

There are two types of VHI in Israel. The first type is VHI offered by public HP to their members (HP-VHI) and covered 88% of the population in 2022 [21]. HPs cannot deny enrollment in HP-VHI, and this type does not require medical underwriting [21]. There are two tiers of HP-VHI, with the higher tier covering more services for higher premiums. Premiums vary slightly by HP but are set based on age in both tiers. These are relatively affordable, ranging from monthly contributions of NIS 6 (~ EUR 1.5) for children to NIS 200 (~ EUR 52) for adults aged 80 + per scheme [24]; benefits are provided primarily in kind. The second type of VHI in Israel is an individual or group policy marketed as several separate schemes by private commercial insurers (C-VHI), which covered 40% of the population in 2022. It requires medical underwriting and can reject applicants [21]. Examples of C-VHI are for pharmaceuticals not covered by NHI, treatments abroad, elective treatment by private providers, and indemnity for severe diseases. Some C-VHI schemes set premiums based on risk, while newer ones set premiums based on age and gender. Additionally, some C-VHI providers do not accept applicants over the age of 65. These plans tend to be more expensive and range from monthly contributions of 20 NIS (~ EUR 5) for children to 217 NIS (~ EUR 56.5) for adults aged 80 + per scheme, adding up to 2000 NIS (~ EUR 500) if several components of insurance are chosen e.g. surgeries and medicines [25]; benefits can be provided both in kind and in cash [26].

Israelis are free to purchase both types of VHI, which result in an extensive dual coverage with about 40% of the population owning both HP-VHI and C-VHI in 2022 [21]. Despite the large share of dual coverage, in case of need, most insured utilize HP-VHI instead of the C-VHI because benefits are easier to receive [27]. As a result, C-VHI is underutilized, even though several reforms have attempted to reduce dual coverage [28].

Given the widespread uptake of VHI and its importance in financing healthcare, it is crucial to understand who utilizes it (and for what type of services), and who does not, to pinpoint potential inequities in access to care among VHI owners. We therefore explored gaps in utilization of HP-VHI, the most commonly owned and used type of VHI in Israel.

Our research questions were:

1. What is the pattern of ownership and utilization of HP-VHI across population groups?
2. Have the rates of ownership and utilization of HP-VHI to finance services changed over a decade (2012–2022)?
3. Which background and socioeconomic characteristics influence the likelihood of using HP-VHI-funded services?

## Methods

### Study design, setting and sampling

We quantitatively analyzed two consecutive cross-sectional data collected through a national survey among those covered by NHI aged 22+. Two rounds of survey were conducted: in 2012 and 2022, among different participants. We performed a secondary analysis of the data collected by the Myers-JDC-Brookdale Institute’s bi-annual surveys on the “Public Opinion on the Level and Performance of Service in the Israeli Health System” [29]. The data collection tool was a closed-ended questionnaire, administered by phone in Hebrew, Arabic and Russian (see Table A1 in the annex for relevant questionnaire prompts).

In 2012, the sampling framework featured computerized telephone lists of landline phone numbers. In each randomly sampled domicile, one resident was further randomly sampled during the phone call to complete the questionnaire. Data were collected from August to December 2012. In 2022, data were collected from October 2021 to March 2022. A random sample was taken from the National Insurance Institute’s database of health plan policyholders. While the second data collection overlap with the COVID-19 pandemic, Israel was a forerunner in vaccination rollout and by early- to mid-2021 utilization and key health expenditure indicators had returned to benchmark levels [30–32]. We therefore assume that the pandemic did not impact the levels of utilization of healthcare by the time of data collection.

Following data collection, the sample was weighted according to population age group, gender, and health plan membership to ensure an accurate representation of the population [33, 34]. We also sought claims data to further validate the self-reported utilization in the national surveys and complement it with granular data on utilization. Yet, the Israeli Health Plans were unwilling to share the relevant information to participate in this research.

### Ethical considerations

This study was ethically cleared by the Myers-JDC-Brookdale Institute’s ethic committee on the 16/08/2021 in accordance with the Declaration of Helsinki. After receiving an explanation about the study and its objectives, participants were requested to provide a verbal consent to participate in the survey. After collection, data was anonymized, and any identifying data was removed.

### Variables and data analysis

Our variables of interests were dummies for reported ownership of HP-VHI (yes/no) among all surveyed participants; and, among owners, reported utilization of HP-VHI to finance groups of services at least once in the two years prior to the survey. We examined the six main service groups covered by HP-VHI: choice of surgeon or hospital; visits to a specialist; dental treatment; prescription drugs not covered by NHI; reproductive health; and child developmental therapies. Bivariate analyses (ꭓ^2^) were applied to estimate the differences in the rate of ownership and utilization of each group of service in 2012 and 2022, and multivariable logistic regressions estimated the odds ratios (ORs) of owning HP-VHI and using each service group at least once during the two years prior to the 2022 survey. We ruled out multicollinearity through Spearman’s correlations significance tests.

We controlled for variables that capture the dimensions that can be used in health inequality monitoring, as defined by WHO [16]: place of residence, race or ethnicity, gender and sex, religion, education, socioeconomic status (SES), and personal characteristics which may be grounds for discrimination or that require adaptations for physical differences (e.g. age, disability). The regression model controlled for health status through the indicators of mental distress, self-reported health status, and chronic disease, which we captured as proxies for need and have been used widely in the literature as such [35–37]. Finally, the model also controlled for HP membership to capture variation in availability and supply of services (full description of the variables available in the annex). The final regression model was:

Logit (P(HP-VHI service_i_ = 1))= β_0_ + β_1_ gender + β_2_ age + β_3_ mental distress + β_4_ self-reported health status + β_5_ chronic disease + β_6_ population group^1^ + β_7_ residence in periphery^2^ + β_8_ HP + β_9_ size of HH + β_10_ education + β_11_ income.

Notes: ^1^ ultra-Orthodox Jews, Arabs, and non-ultra-Orthodox Jews; ^2^ Residence in a peripheral or highly peripheral city is defined by the Central Bureau of Statistics, as relative to residence in a non-peripheral city. The periphery index is a weighted average of two components: potential for access, and the proximity to the border of the Tel Aviv Region. The values of the index range from 1 to 10, and cities with a value of between 1 and 4 are defined as peripheral or highly peripheral.

## Results

### Final sample and representativeness

In 2012, 2,330 individuals aged 22 + were interviewed, with a response rate of 61%; while in 2022 the response rate was 52% with 2,536 individuals aged 22 + interviewed. Both samples represent Israel’s population according to background and SES variables, and health status. Table 1 presents the characteristics of those sampled in the 2012 and 2022 surveys compared to the population data in 2022.

**Table 1:** Socio-demographic characteristics of the survey samples (2012 and 2022) and general population, 2022 (%)

Variable (%)	2012 survey^	2022 survey^	General population (2022)^^
	*N* = 2,330	*N* = 2,536	
Women	52	51	51
Age			
22–34	21	29	29
35–44	22	20	20
45–54	24	17	17
55–64	17	14	14
65+	16	20	20
Population group			
Jews	83	81	81
Of those, ultra-Orthodox	7	9	11
Arabs	17	19	19
Residence in peripheral area	15	15	19
Health plan			
Clalit	53	52	52
Maccabi	25	28	28
Meuhedet	14	12	12
Leumit	8	7	7
Chronically ill	30	34	-
Income in the lower quintile^^^	20	21	20
Academic education	40	47	34

Notes: ^ Data presented are data for people aged 22 and over, weighted, by year. For more information, see the methodological appendices (2022: https://brookdale-web.s3.amazonaws.com/uploads/files/Methodological_Appendix.pdf; 2012: https://brookdale-web.s3.amazonaws.com/uploads/2018/01/705-15_Methodology_Document.pdf). ^^ Sources for gender, age, nationality, health fund district – from up-to-date information received from the National Insurance Institute in interpersonal communication by correspondence from August 2022; ultra-Orthodox Jewish population over the age of 20 [38]; Education Data [39]; Peripherality: Peripherality Index of Localities and Local Authorities, 2020 [40]; ^^^ In the 2012 survey, income was calculated as standard gross per capita income in the previous month; And all survey respondents were divided into quintiles

### Reported ownership of HP-VHI

**Fig. 1:**
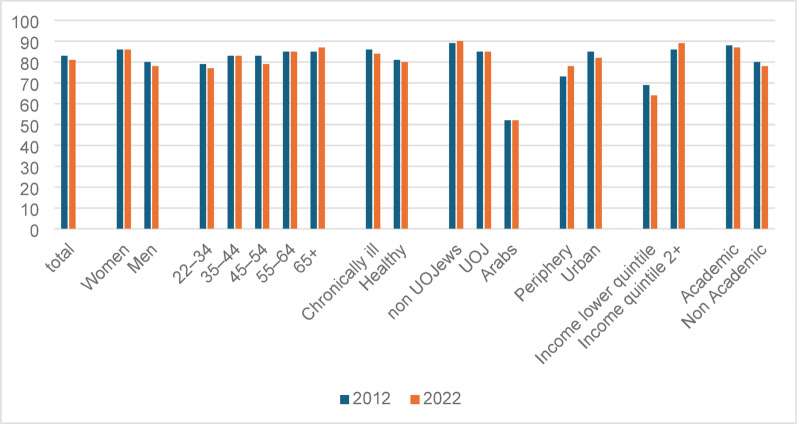
Rates of reported HP-VHI ownership, by demographic SES characteristics, and chronic illness (%). UOJ - ultra-Orthodox Jews; the reported rates of HP-VHI ownership vary from those published elsewhere as here they cover only adults aged 22 + and not the entire population

**Fig. 2:**
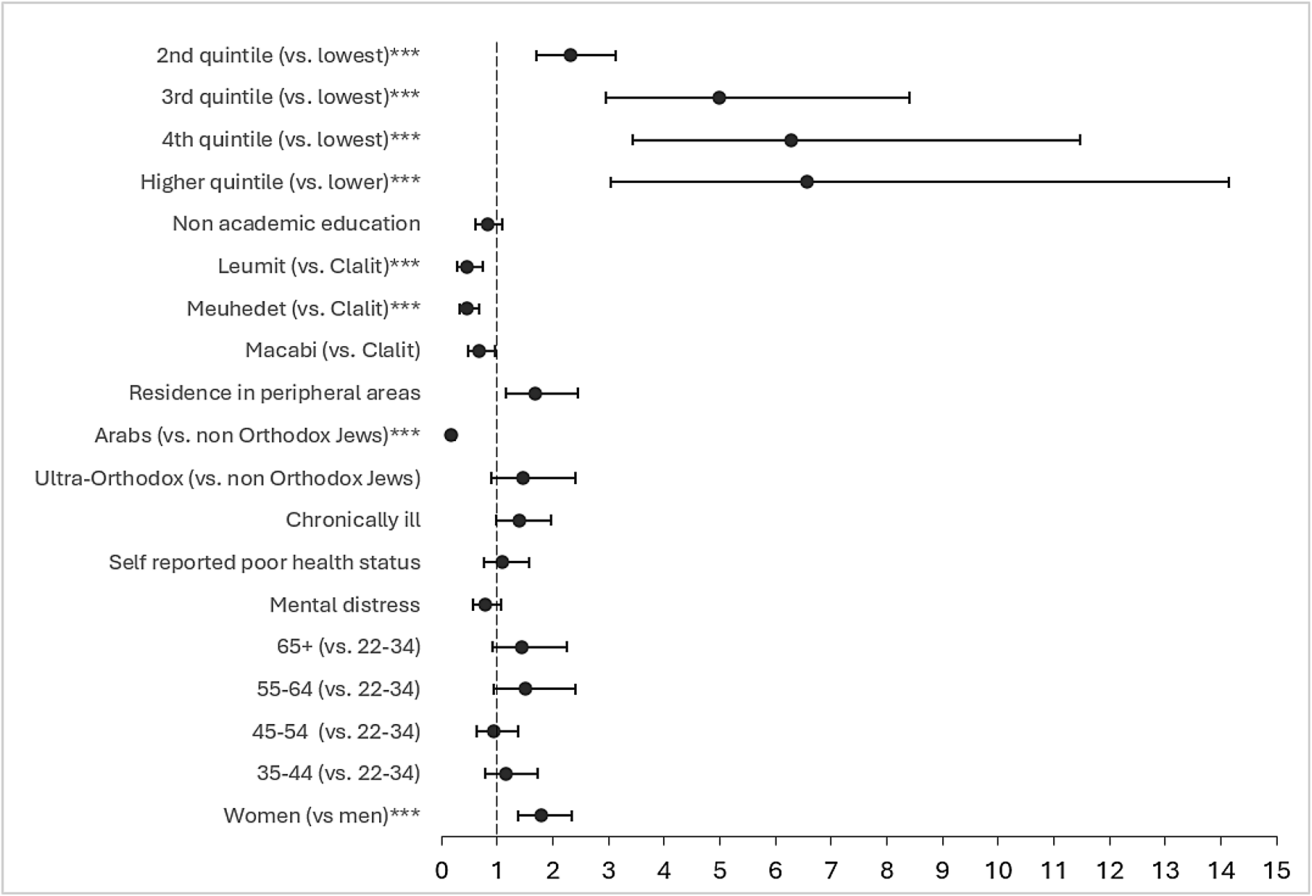
Multivariable logistic regression: ORs (and confidence intervals) of reported owning HP-VHI by background characteristics, 2022. Notes: *p < 0.05; **p < 0.01; ***p < 0.001; for the full results of the regression model see Table A2 in the annex. Quintiles refer to income quintiles

First, we identified gaps in reported HP-VHI ownership. We disaggregated the rates of reported ownership by population group in the two survey rounds (Fig. 1) and explored the odds of reported owning HP-VHI in 2022 (Fig. 2).

The rate of total reported HP-VHI ownership (82%) remained similar in the two surveys. Women, older adults (over 55) and chronically ill individuals reported higher rates of ownership in both rounds. Arabs reported lower rates of ownership (52% in both years), as well as those living in peripheral areas (73% in 2012, 78% in 2022) and those with income in the lower quintile (69% in 2012, 64% in 2022). The multivariable analysis with data from the 2022 survey revealed that women are more likely than men to report owning HP-VHI, as well as those with income above the lowest quintile (Fig. 2). Compared to non-ultra-Orthodox Jews, Arabs are less likely to report owning HP-VHI, while ultra-Orthodox Jews are more likely than non-ultra-Orthodox Jews to report owning it; as well as members of Leumit and Meuhedet health plans (compared to the biggest HP, Clalit).

### Reported utilization of HP-VHI

**Fig. 3:**
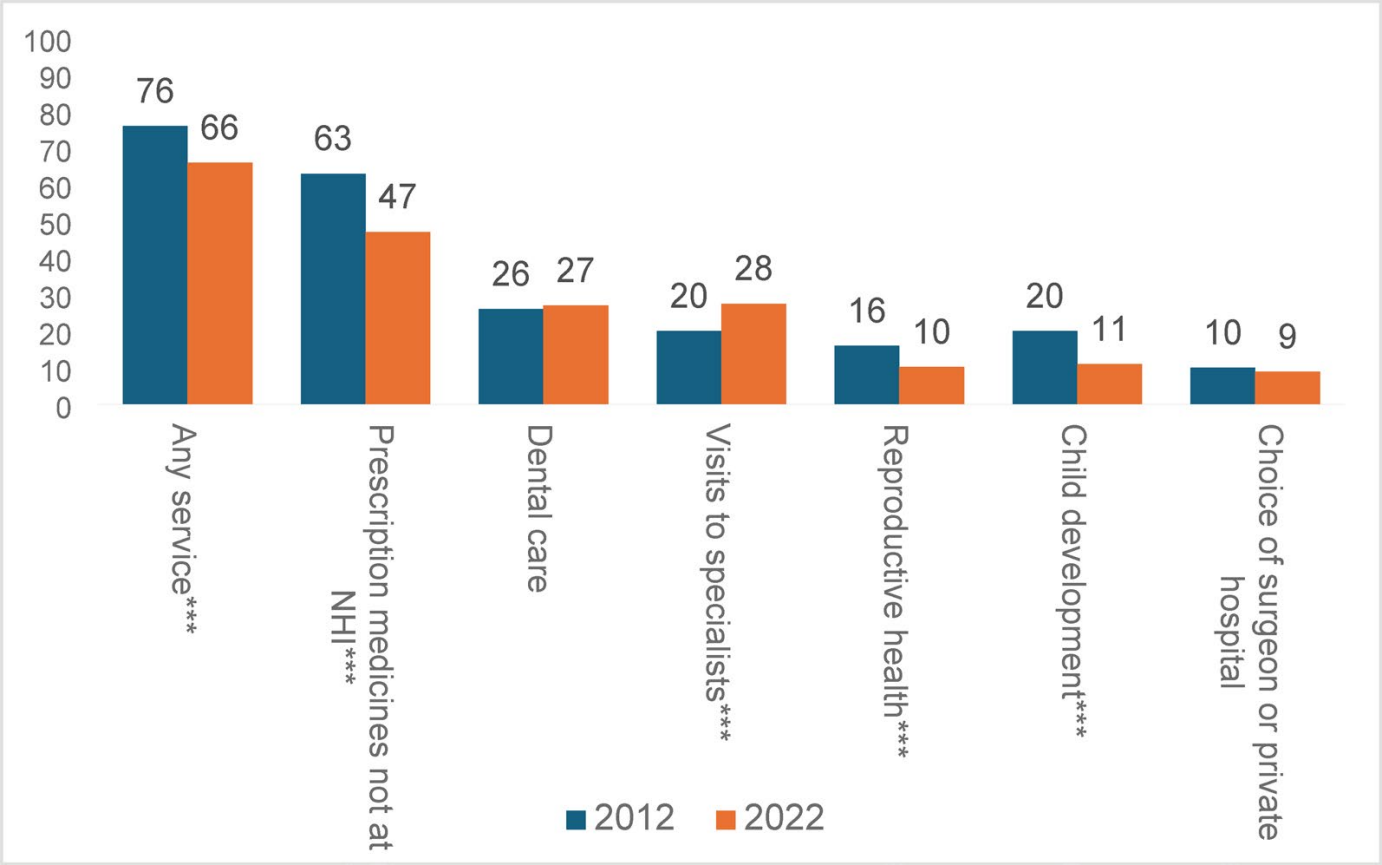
Rates of reported utilization of at least one service among HP-VHI owners during the two years preceding the surveys (%). *p < 0.05; **p < 0.01; ***p < 0.001.

The 82% who reported owning HP-VHI were asked whether they had used it to finance each of the six groups of services at least once in the two years prior to the survey. Total reported utilization declined from 76% in 2012 to 66% in 2022 (Fig. 3). After a decade, reported utilization of HP-VHI declined for most groups of services, except for visits to specialists, which increased from 20% to 28%. Of all services, the highest reported utilization was for purchasing prescription medications not covered by NHI, though this declined from 63% in 2012 to 47% in 2022. Dental care and choice of surgeon remained unchanged, with about 27% and 10% of participants reporting utilization in the last decade.

**Fig. 4:**
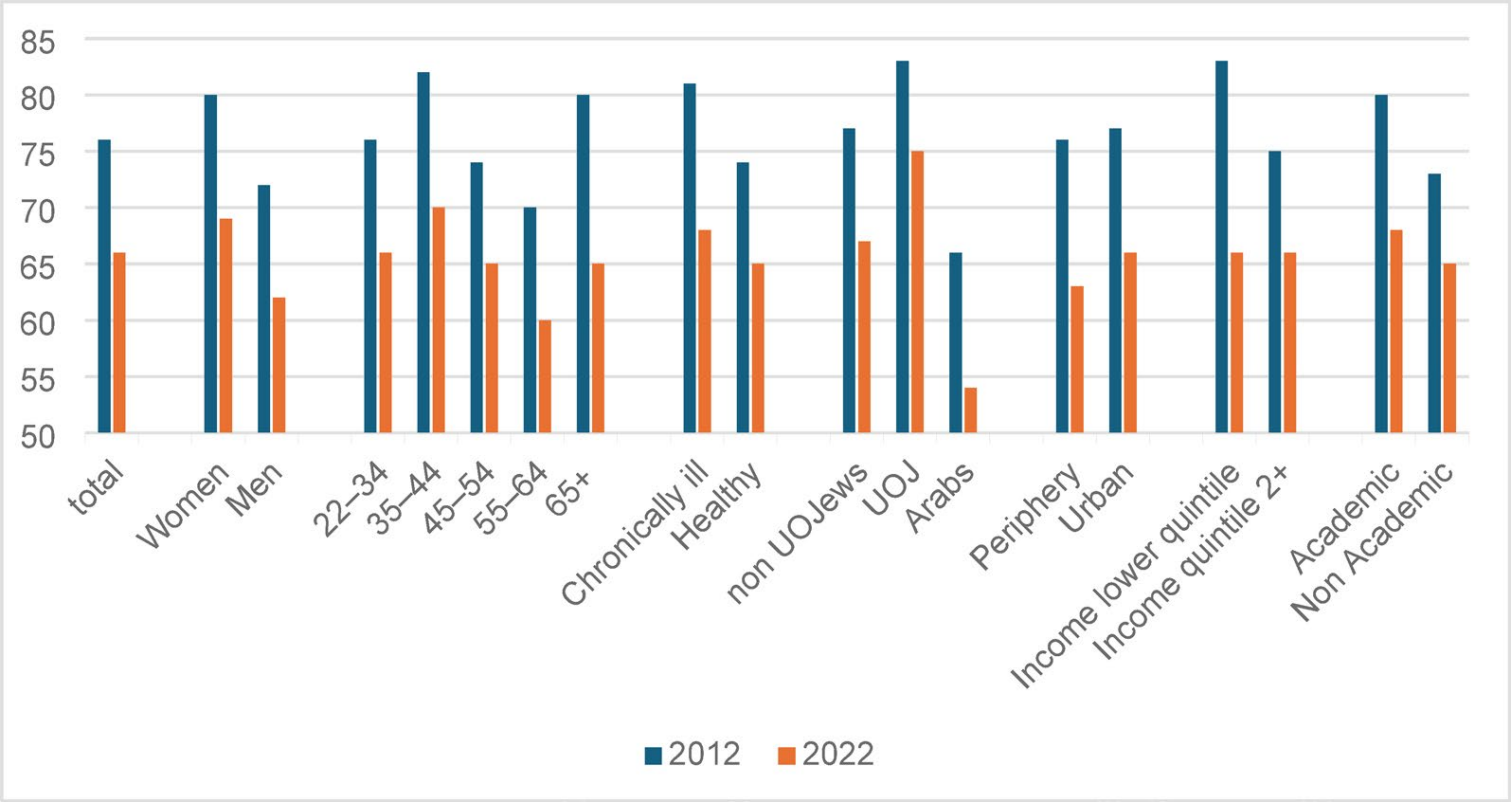
Rates of reported utilization of at least one service among owners during the two years preceding the surveys, by demographic and SES characteristics (%). The difference between reported utilization rates was tested with a chi-square test, and is significant at a *p* < 0.01 between 2012 and 2022, and across all variables.

When disaggregating rates of reported utilization of HP-VHI by population group, men and Arabs reported lower than average rates both in 2012 and in 2022 (Fig. 4). While in 2012, ultra-Orthodox Jews and people with income in the lower quintile reported higher utilization (83% compared to the average of 76%), in 2022 those with low incomes reported average utilization rates; ultra-Orthodox Jewish utilization declined to 77%. Between 2012 and 2022 reported HP-VHI utilization declined more sharply than the average among men, adults older than 55, those living in peripheral areas, and those in the lower income quintile (about 20% compared to the average of 12%).

Table 2 presents the ORs of HP-VHI owners’ reported utilization of HP-VHI to finance each group of services separately in 2022. HP-VHI owners with higher incomes, higher education and living in central areas of the country are more likely to report using HP-VHI than others. While those with income in the fourth and highest quintile are more than twice as likely (ORs 2.28 and 2.87, respectively) to report utilization of HP-VHI to visit a specialist than those in the lowest income quintile, those with non-academic education and those living in peripheral areas are less likely (ORs 0.68 and 0.64) than those with academic education and those living in non-peripheral areas of report using the same services. Those with higher income quintile are more than 4 times more likely to report utilization of HP-VHI to finance reproductive health services than those in the lowest income quintile.

People with income in the lowest quintile are more likely to report using HP-VHI to pay for medicines. After controlling for all other variables, Arabs are more likely than non-ultra-Orthodox Jews to report using HP-VHI for purchasing medicines (OR 1.51), choose a surgeon or private hospital (OR 2.68), and reproductive health (OR 3.20). Compared to other Jews, ultra-Orthodox Jews are more likely to report using HP-VHI to pay for medicines (OR 2.13), choose a surgeon or private hospital (OR 2.24), and for child development services (OR 2.17).

Health status, as captured by chronic illness and mental distress, does not signficantly influece HP-VHI reported utilization. However, self-reported poor health status is associated with higher likelihood of report using HP-VHI for choosing a surgeon or private hospital (OR 1.63), visiting a specialist (OR 1.70) or purchasing medicines (OR 1.61). Age, sex and HP also influence the likelihood of report using HP-VHI in diverse directions across the different groups of services.

**Table 2:** Odds ratios for reported utilization of each group of services by population group, 2022

	Choice of surgeon or private hospital OR (CI)	Visit to a specialist OR (CI)	Dental care OR (CI)	Child development OR (CI)	Outpatient prescription medicines OR (CI)	Reproductive health OR (CI)
Women (vs. men)	1.67**	1.24	1.06	*n*/a	1.27*	*n*/a
	(1.15–2.44)	(0.97–1.59)	(0.83–1.37)		(1.01–1.6)	
Age group						
35–44 (vs. 22–34)	0.80	1.54*	0.88	n/a	1.12	0.51
	(0.44–1.47)	(1.05–2.25)	(0.62–1.25)		(0.79–1.59)	(0.25–1.07)
45–54 (vs. 22–34)	1.00	1.67**	0.92	n/a	0.76	0.26**
	(0.54–1.82)	(1.13–2.47)	(0.64–1.33)		(0.53–1.08)	(0.10–0.69)
55–64 (vs. 22–34)	0.74	0.76	0.79	n/a	0.42***	n/a
	(0.38–1.44)	(0.49–1.20)	(0.53–1.18)		(0.28–0.61)	
65+ (vs. 22–34)	1.77*	1.45	0.44**	n/a	0.59**	n/a
	(1–3.11.11)	(0.97–2.17)	(0.26–0.74)		(0.41–0.85)	
Mental distress	1.31	0.93	1.09	n/a	1.24	0.56
	(0.86–1.99)	(0.69–1.26)	(0.79–1.49)		(0.94–1.64)	(0.23–1.36)
Self-reported poor health status	1.63*	1.70**	0.95	n/a	1.61**	0.61
	(1.03–2.56)	(1.21–2.38)	(0.64–1.39)		(1.17–2.22)	(0.19–1.98)
Chronically ill	1.45	1.09	0.75	n/a	1.31	1.41
	(0.93–2.26)	(0.80–1.48)	(0.54–1.04)		(0.99–1.75)	(0.60–3.3)
Population group						
ultra-Orthodox (vs. non-ultra-Orthodox Jews)	2.24**	1.35	1.21	2.83**	2.13***	1.27
	(1.20–4.19)	(0.85–2.17)	(0.77–1.89)	(1.49–5.4)	(1.38–3.29)	(0.34–4.71)
Arabs (vs. non-ultra-Orthodox Jews)	2.68***	0.81	0.83	1.18	1.51*	3.22**
	(1.48–4.85)	(0.49–1.32)	(0.53–1.30)	(0.60–2.32)	(1.01–2.25)	(1.31–7.9)
Residence in peripheral areas	0.76	0.64*	1.16	0.53	0.92	1.66
	(0.43–1.36)	(0.42–0.98)	(0.79–1.69)	(0.26–1.09)	(0.65–1.29)	(0.73–3.76)
Health plan						
Macabi (vs. Clalit)	0.94	0.73*	1.48**	0.61*	1.00	0.42**
	(0.61–1.46)	(0.55–0.98)	(1.11–1.98)	(0.37–1.01)	(0.77–1.31)	(0.18–0.95)
Meuhedet (vs. Clalit)	1.07	0.94	0.95	0.44*	1.01	0.81
	(0.61–1.91)	(0.63–1.41)	(0.62–1.45)	(0.22–0.91)	(0.69–1.48)	(0.28–2.31)
Leumit (vs. Clalit)	1.26	1.19	0.78	1.04	1.19	1.37
	(0.64–2.49)	(0.73–1.96)	(0.44–1.36)	(0.48–2.28)	(0.74–1.91)	(0.42–4.44)
Size of Household	0.96	0.93*	0.98	1.07	0.96	0.79*
	(0.87–1.08)	(0.86–1.00.86.00)	(0.91–1.05)	(0.96–1.19)	(0.90–1.03)	(0.65–0.96)
Non-academic education	0.86	0.68**	1.11	0.78	1.02	0.77
	(0.58–1.28)	(0.52–0.89)	(0.84–1.46)	(0.49–1.24)	(0.80–1.3)	(0.39–1.55)
Income quintile						
Higher (vs. lowest)	1.73	2.87***	0.76	1.08	0.48**	4.31*
	(0.77–3.91)	(1.66–4.96)	(0.43–1.34)	(0.43–2.68)	(0.28–0.81)	(1.03–17.98)
4th (vs. lowest)	1.47	2.28***	0.99	1.43	0.63*	2.45
	(0.74–2.92)	(1.41–3.69)	(0.61–1.60)	(0.63–3.26)	(0.41–0.96)	(0.69–8.64)
3rd (vs. lowest)	1.31	1.78**	1.07	0.87	0.60*	2.01
	(0.69–2.50)	(1.12–2.83)	(0.68–1.71)	(0.37–2.04)	(0.40–0.91)	(0.57–7.01)
2nd (vs. lowest)	1.20	1.40	1.09	1.24	0.59**	1.37
	(0.72–2.02)	(0.94–2.08)	(0.74–1.61)	(0.61–2.52)	(0.42–0.83)	(0.48–3.88)
Constant	0.04***	0.25***	0.41***	0.12***	1.23	0.28*
Nagelkerke R Square	0.08	0.09	0.04	0.07	0.09	0.15
N	1461	1414	1266	743	1312	508
Filter^1^			age < 72	age < 55 and has children > 1		women = 1 and age < 45

Numbers in bold are significant at *p < 0.05; **p < 0.01; ***p < 0.001

Note: 1 For ‘dental care’ individuals aged 72 or more were excluded, as this group is covered by NHI and not entitled to this service through the HP-VHI. In ‘reproductive health’ services, we excluded men and individuals over the age of 55, as they are not covered. For ‘child development’ services we excluded individuals who did not report having children

## Discussion and policy implications

Our study adds to the limited body of evidence examining the equity of VHI as a source of health financing by exploring gaps in reported ownership and reported utilization [12]. We found that in Israel, rates of reported ownership vary by population group, with those with higher SES reporting higher rates of ownership. This is similar to the well documented VHI market in several other countries [11, 41–44]. While in Israel the rate of reported ownership of HP-VHI remained stable between 2012 and 2022, the reported rate of utilization of HP-VHI to pay for at least one group of services was lower in 2022 compared to 2012. Rates of reported utilization declined more among men, older adults, and Arabs – the very same groups that also report lower rates of owning HP-VHI. In addition, those with lower incomes, lower levels of education, and residents of peripheral areas are less likely to report using HP-VHI to pay for certain groups of services. This may reflect financial barriers to accessing HP-VHI funded services, probably due to user charges policies, which do not have protection mechanisms for vulnerable populations (as the NHI does), or due to lower availability of care in their place of residence [45]. Moreover, these are the very groups who typically face access barriers and report forgoing publicly funded care [33]. It seems that HP-VHI is not improving access to care to these groups, as expected. In addition, lower utilization of HP-VHI funded services is associates with lower health insurance literacy [46]. While measuring health insurance literacy is not easy[47], evidence shows that Arabs report lower levels compared to Jews [48]. Finally, respondents from a high SES might have reported higher rates of HP-VHI utilization because they are typically covered by the higher tier of HP-VHI, which offers a wider range of services.

Since VHI has been an important source of financing healthcare in Israel in the last two decades, the fact that reported utilization of HP-VHI has dropped between 2012 and 2022 for most services indicates that fewer insured are benefiting from coverage. This is particularly true for insured people from lower SES, which are the very groups that could benefit from this extra layer of coverage.

### The interplay between mandatory and voluntary health insurance raises important policy questions

VHI is scrutinized by its role in relation to public coverage. VHI can fill gaps or expand the public coverage by complementing it (covering user charges of covered services), supplementing it (with services not covered), substituting it (covering people otherwise not covered by the public coverage) or duplicating it (providing better access and more choice of services already publicly covered) [8, 49]. VHI in Israel supplements and duplicates NHI, and owners expect it to reduce waiting times and enhance choice of provider [20, 26]. Within its supplementary role for services not publicly covered, VHI ownership leads to less utilization inequality than if there were no VHI. In this sense, VHI partially fills the public coverage gap, even if it is in an unequal manner.

Given that a large share of the Israeli population owns HP-VHI, it is important for stewards and stakeholders of the Israeli health system to define what its role should be in covering services vis-a-vis the public insurance system. With the presence of both HP-VHI and C-VHI, as well as the fact that nearly one in four individuals have double coverage, the interplay between the public and private systems should be clarified to avoid the continuous creep of VHI ownership contributing to inefficient health system financing. Our findings emphasize the need to unveil this interplay. For example, do our findings of low reported HP-VHI utilization mean good coverage and access to services covered by the public benefits basket, or rather low access to public and private health services? Should policymakers interpret the findings of older adults’ lower reported utilization of HP-VHI to finance medicines as them having adequate coverage and access to medicines financed by the public benefits basket, with proper protection mechanisms in place (e.g. discounts, exemptions and caps)? Or does the lower reported utilization indicate financial or other access barriers for medicines covered by both insurances? Another issue to consider is the exacerbation of a two-tiered system, whereby those insured benefit from better access and quality of care [50, 51].

Another important policy question is the role of HP-VHI in filling the gaps in public coverage. Should it expand public coverage with nice-to-have, but not essential services such as alternative medicines or cosmetic surgery? Or should it improve access to essential services such as outpatient specialist care? If so, is HP-VHI the most efficient source of financing essential services?

While Sect. 10 of the NHI law states that HP-VHI should not include services covered by the NHI (except dental care – which is currently not covered by the NHI), Sect. 10 does not clearly define the role of HP-VHI. In practice HP-VHI duplicates several of the NHI-covered services with broader choice and faster access to care. In addition, despite that the Ministry of Health regulates HP-VHI and regularly publishes circulars and procedures regarding the mode of operation of HP-VHI, it has not yet defined explicitly HP-VHI’s main role. The absence of a clear strategy and a clear definition of the goals and purposes of the HP-VHI (in the various tiers) leads to uncertainty and inefficiencies in the HP-VHI market, potentially undermining access to covered services. There is a real need to formulate a clear policy and tighten the regulation on HP-VHI programs – both regarding the composition of the coverage offered in each of the HP-VHI tiers and its accessibility to all insured.

Having said that, the MoH has been attempting to regulate the HP-VHI market for over a decade, aiming at mitigating some of these inefficiencies. For example, to make HP-VHI more accessible, eligibility waiting periods for people switching HP-VHI plans due to changes between HPs were abolished in 2018 [21, 23]. To limit the costs of services, and consequently the level of premiums, since 2011, HP-VHI was no longer allowed to cover services provided by professionals without contracts with insurers [52]. To limit the duplicative role of HP-VHI, in 2008 the MoH reinforced the prohibition to cover life-saving/extending medications. And to limit the levels of user charges, payments for surgeries (including physicians’ fees) changed between 2015 and 2017 to be made to hospitals, with no direct payment to physicians [26]. More recently, the MoH proposed excluding “services with no significant medical value” from the HP-VHI services, yet this is still under debate. Nevertheless, these regulations still do not specify the roles of HP-VHI and do not successfully mitigate the inequities in access among HP-VHI owners.

### VHI as an imperfect financing policy to substitute NHI (public) funds

Public coverage typically has three main roles: (1) Insuring, meaning reducing households’ financial risk associated with illness; (2) Providing access to needed health services (by reducing financial barriers), and (3) Equity, by redistributing funds from rich to poor and healthy to sick [45]. In principle, VHI could perform these roles and could be an alternative source of prepaid funds to cover the gaps of public coverage, particularly as public funds are stretched.

First, as an insurance, VHI pools prepaid funds and spreads the risks and costs of illness over time and across people. It reduces households’ financial risks associated with illness and is a better source of funding than OOP payments. Second, in terms of access to care, in principle, duplicative and complementary VHI can enhance access to and choice of provider. However, HP-VHI in Israel does not seem to be filling these functions by protecting owners from financial or geographic access barriers to publicly financed healthcare. As we show, HP-VHI owners with low SES still report lower rates of utilization of VHI, and evidence from other surveys show that HP-VHI owners report average rates of unmet needs due to cost or distance [21, 33].

Second, regarding access, in principle, VHI improves access, expands choice, and provides an extra layer of financial protection from costs. Policymakers could consider VHI as an alternative source of funds to the stretched public coffers. However, as in other countries, HP-VHI in Israel is a regressive funding mechanism, with premiums calculated based on age – a proxy for health needs or risks rather than financial capacity. Therefore, VHI is not a fair and equitable source of funds [53].

VHI is, nevertheless, an instrument for reducing waiting times for those who own it. VHI owners report higher-than-average rates of unmet needs for publicly financed healthcare due to waiting times; with half of them skipping queues in the public system by seeking and getting care privately funded [54]. In this case, duplicative VHI contributes to enhanced access to certain services for many people, improving overall population well-being. At the system level, societies and policymakers have to weigh these benefits against the increased inequities that result between VHI owners and non-owners, among owners, and between those with high and low SES.

In addition, the hypothesis that VHI could generate health benefits both for owners and non-owners through the “substitution effect” where VHI owners seek private care, “freeing up” capacity in the public system for the non-owners is not supported by evidence. A recent study found that in European countries and Israel, increases in VHI coverage rates were linked to a long-term rise in the prevalence of poor health, which is more consistent with the crowding-out hypothesis (in which VHI divert resources away from the public system, lowering the quality of public health services and consequently leading to negative health outcomes for those who remain dependent on the public sector). These findings were more pronounced in countries with duplicative VHI, and among people with low SES [55].

Third, VHI could also promote equity. If the poor or the sick used VHI more than others to pay for care, funds would be redistributed from the rich to the poor and from the healthy to the sick [12]. In Israel, this secondary (or even tertiary) coverage layer often benefits just a portion of the population who can afford to purchase VHI —typically wealthier and healthier individuals. Multiple coverage results in a two-tiered system where VHI ownership is related to income, and one that intensifies existing healthcare inequalities and less financial protection on the population level [11, 55, 56]. Our findings show that HP-VHI does not promote equity among owners, as those with lower SES report using certain services less than others, after controlling for health status. The fact that reported utilization of HP-VHI is lower for certain services among those with low SES (low incomes, low education and residency in the periphery) can be interpreted as HP-VHI being a poor policy tool to protect these populations from access barriers. Moreover, in practice, they subsidize insured people with high income, high education and residents of central areas.

HP-VHI does not perform two of the three roles of public insurance, and therefore, is not an alternative source of financing healthcare. Our study underlines that financial protection and equity are not the primary goals of VHI in practice. While VHI may increase access to care, its focus is generally on profitability and serving individuals who can afford premiums, rather than ensuring access or protecting vulnerable populations from high healthcare costs. While there are policy tools to mitigate regressivity of VHI, it widens disparities in access between owners and non-owners [8, 42, 55, 57]. We also show that inequalities in reported utilization of HP-VHI also persist among owners. Policymakers aiming to have an equitable and fair financing mechanism for health services should refrain from VHI as an alternative source.

In most HICs, including Israel, public funds promote efficiency, as they cover technologies and services deemed essential and cost-effective after being assessed by a systematic and transparent process. VHI is less efficient, as it may also cover non-essential and non-cost-effective technologies and services, providing lower-value care compared to public coverage. It is also often considered a luxury good in countries with universal coverage [58]. In addition, public funds are typically proportional or progressive, but rarely regressive [13, 59, 60]. Progressivity could be improved if the funds allocated to VHI were collected as contributions for the NHI and expanding its coverage and access [21, 27]. This, however, is a long-running challenge, as VHI is a popular policy option in times of rising prices, inflation and competing budgetary obligations for governments, even if it is likely not a sustainable financing option over the long term [61–64].

### Limitations

Due to its exploratory nature, this study has several limitations. The main limitations of our study relate to the phone surveys upon which the analysis is based. Our study relies on self-reported data, which may be subject to recall bias or social desirability bias. Respondents may report inaccurate information due to forgetting specific instances of usage or due to misinterpretation of coverage. Additionally, as HP-VHI ownership in Israel is so ubiquitous, it is possible that respondents mistook which type of insurance they used for a service. Furthermore, it is unknown to what extent insured residents in Israel know that they own (either HP-VHI, C-VHI, or both) and what benefits it gets them versus just relying on their public coverage. However, to address this issue, when asking about HP-VHI ownership, we provided clear examples by naming the HP-VHI insurers to avoid ambiguity. Additionally, when inquiring about the utilization of HP-VHI to pay for services, we asked about the level of copayments, which are clearly higher than those charged by the NHI, to avoid this ambiguity.

Second, the weighting applied post-data collection did not account for population group (e.g., Arabs, ultra-Orthodox Jews) or region. Since the analysis focuses explicitly on these groups, omitting them from the weighting process could have led to underrepresentation or bias. However, Table 1 shows that weighting for age, gender, and health plan was enough to reach a representative sample in both waves of the survey.

A third limitation relates to the limited data collected. Our study does not capture the frequency, intensity, or associated costs related to reported utilization of HP-VHI to pay for each group of services, as the data is limited to a binary reported ‘used/not used’ response. This reduces meaningful variability as well as the ability to examine marginal effects on demand or elasticity, repeated use, catastrophic expenditures, or behavioral responses to insurance design. Furthermore, our estimations of access to care funded by HP-VHI are limited, particularly because the costs and frequency of use of each service group vary. Our findings should be complemented with claims data (which were not made available upon request) that can assess more precisely the types of services used with VHI (e.g. chronic versus episodic use, medications, etc.), to understand patterns of frequency and costs and distribution of financial burden. These additional analyses will help to assess whether VHI is equally utilized and financed and further determine whether services utilized through VIH are “nice to have” or “essential”. Claims data, however, may not contain individual information such as health status, population group, education or income, which we were able to capture in our survey; these data were important to assess individuals’ background and socioeconomic variables associated with realized access of VHI-funded care.

Fourth, our multivariable models presented low explanatory power (the Nagelkerke R² values reported in the logistic regressions are relatively low (0.04–0.15)), suggesting much variance remains unexplained. Other potentially relevant and access-related variables, such as provider availability (especially in the public system), waiting times, linguistic barriers, awareness of entitlements, health literacy, or trust in providers, could have been added to the model to explain utilization of VHI-funded care. However, the objectives of our work were to assess background and socioeconomic characteristics associated with realized utilization and access, not fully explain the reasons behind utilizing VHI to fund care. Therefore, adding these variables and others was out of scope.

Fifth, we did not directly ask about VHI access barriers such as forgoing VHI-funded care due to user charges or distance to provider. This may have impacted our understanding of financial and geographical barriers to accessing care, particularly for those with HP-VHI coverage. This information can be complemented by qualitative future studies.

Finally, we did not complement our findings with data on availability of care, public or private. This information could have provided a more complete picture regarding the interplay between private and public insurance markets, and the reasons for utilizing (or not) VHI. However, this information is not publicly available and collecting it was beyond the scope of our work.

## Conclusions

With public sources of funds for health under pressure, policymakers can be tempted to use VHI as an alternative source of prepaid, pooled funds to cover the gaps in public coverage. However, VHI is not a viable or efficient alternative. Not only is VHI a regressive financing source – it creates access gaps between the insured and uninsured, but also between the insured, and gaps in access persist based on income, education, and residence in the periphery. VHI provides access mainly for those who already have better access, not those with higher needs or lower capacity to pay for healthcare.

## Supplementary Information

Supplementary Material 1

## Data Availability

Data is not publicly available due to privacy and anonymization requirements. Aggregated data can be found at “Trends in public opinion about the level of service in the health system and its functioning, according to background characteristics – an interactive report”. Jerusalem: Myers-JDC-Brookdale Institute, 2025. ([https://brookdale.jdc.org.il/en/publication/trends-in-public-opinion-about-the-level-of-service-in-the-health-systeminteractive-report/](https:/brookdale.jdc.org.il/en/publication/trends-in-public-opinion-about-the-level-of-service-in-the-health-systeminteractive-report)).

## Abbreviations

CBS: Central Bureau of Statistics
CHE: Current health expenditures
C: VHI–commercial voluntary health insurance
EUR: Euros
HIC: High income country
HP: Health plan
HP: VHI–health plans’ voluntary health insurance
LMIC: Low–and middle–income country
MoH: Ministry of Health
NHI: National health insurance
NIS: New Israeli Shekels
OOP: Out–of–pocket
ORs: Odds ratios
SES: Socio–economic status
UOJ: Ultra–orthodox Jews
VHI: Voluntary health insurance

## Declarations

and ethics statements.
