# Supplementary material for: Insured but unequal: who really utilizes voluntary health insurance in Israel? Evidence from two consecutive cross-sectional studies
Source: Isr J Health Policy Res. 2026 May 29;15:22. doi: 10.1186/s13584-026-00763-2 (PMC13220429; doi:10.1186/s13584-026-00763-2)
Supplement: Supplementary file 1 — Supplementary Material 1 [file 13584_2026_763_MOESM1_ESM.docx]

## Annex A1 Description of variables

### Dependent variable

**Utilization of HP-VHI services.** The participants who have HP-VHI, were asked about the utilization of HP-VHI in the two years preceding the survey. HP-VHI services examined: choosing a surgeon and a private hospital, visiting a consultant doctor, discounting dental care in clinics, child development services (e.g., occupational therapy, speech therapist, etc.), discount on the purchase of medications as well as fertility treatments, pregnancy and childbirth (for details of the survey questions see Table A1).

For each service, a dummy variable was built with two categories: "used the service in the last two years" and "did not use the service in the last two years." In other words, there are six dummy variables for VHI services. A unified variable was also built, in which the question "Have you used at least one service in the past two years?" was coded.

#### Table A1: Questionnaire questions

| 2022 Survey | 2012 Survey |
| --- | --- |
| I will now read you a list of services that can be obtained through the HP-VHI. In the past two years, have you used the following services, through the HP-VHI: | I will now read you a list of services that can be obtained through HP-VHI. In the past two years, have you used the following services, through the HP-VHI: |
| 52.1 Surgery in a private hospital or choosing a surgeon (for interviewers: or private medical services in Jerusalem) | 53.1 Private Surgery and Surgeon |
| 52.2 A visit to a doctor or other professional and you paid more than 30 shekels | 53.2 Second expert opinion |
| 52.3 Discount for Dental Care | 53.3 Discount on Dental Care |
| 52.4 Child development services, for example, occupational therapy, speech therapist, and you paid more than 30 shekels. | 53.4 Child treatments (e.g., occupational therapy, speech therapist) |
| 52.5 Discount on the purchase of medications | 53.5 Discount on the purchase of medications |
| 52.6 [For interviewers: ask only women] Fertility and pregnancy treatments beyond the benefits basket | 53.6 [For Interviewers: Skip Men to Question 53.7.] Women and Pregnancy (Including Fertility Treatments Beyond the benefits Basket) |

### Independent variables

**District of residence**. North, Haifa, Center, Tel Aviv, Jerusalem and West Bank (Judea and Samaria) and South.

**Residence in the periphery.** Dummy categorical variable that divides the sample into residents of peripheral areas and residents who live elsewhere. This variable is based on a peripherality index developed by the Central Bureau of Statistics, and it ranks local authorities in Israel according to their geographic location relative to population centers. The value of the index is calculated as a weighted sum of two components: (1) potential accessibility, and (2) proximity to the Tel Aviv district border.^[[1]](#footnote-1)^ The respondents' residential localities were translated into the value of their peripheral index: localities in clusters 1-4 were classified in the category "periphery" (according to the Central Bureau of Statistics definition, they are considered "peripheral" and "very peripheral"), localities in clusters 5-10 were classified as "non-peripheral" (according to the Central Bureau of Statistics they are considered "medium" in their peripheral degree, "central" and "very central"), (Central Bureau of Statistics, 2008).

**Chronic illness.** Dummy variable with two categories: 'there is a chronic disease' and 'there is no chronic disease'.

**Emotional distress.** A dummy variable with two categories: 'has felt emotional distress in the past two years' and 'has not felt emotional distress in the past two years'.

**Subjective state of health.** Each interviewee rated his general health on one of five options: very good, good, medium, not good, and poor. The interviewee's subjective perception of his general health was coded into a dummy variable: 'good health' which contained the interviewees who answered 'very good' and 'good', and 'not good health', which contained the answers 'moderate', 'not good' and 'poor'.

**Income.** The sample was divided into five quintiles based on the question: "Last month, what was your total net income and that of all family members living with you, from all sources of income? This refers to income from work, pensions, allowances, rent or any other fixed source of income. Within the ranges I will read, please stop me in the appropriate category." This question has low answer rates (22% missing entries). Missing values were completed using the median.

**Education.** A variable with two categories: 'academic education' and 'no academic education'.

**Population group.** Variable with three categories: 'non-ultra orthodox Jews', ' ultra orthodox ' and 'Arabs'. The Central Bureau of Statistics divides Israel's population into three categories: Jews, Arabs (Muslims, Arab Christians, and Druze) and others (non-Arab Christians, members of other religions, and people who were not classified in the population registry as Jews, Muslims, or Christians), but in this study "ultra-Orthodox" and "non-Haredi Jews" were also referred to as "population groups."

**Number of household members**. Proxy for multiple children.

**Age.** Categorical variable with five levels: (1) age 22–34; (2) age 35–44; (3) Age 45–54; (4) Age 55–64; (5) Age 65 or older.

**Sex.** A variable with two categories: 'man' and 'woman'.

## Table A2: Multivariate logistic regression: odds ratio of owning VHI by background characteristics, 2022

| Variables | Exp(B) | 95% C.I.for EXP(B) | |
| --- | --- | --- | --- |
|  |  | Lower | Upper |
| Women (vs men)*** | 1.796 | 1.373 | 2.348 |
| Age |  |  |  |
| 35-44 (vs. 22-34) | 1.161 | 0.784 | 1.718 |
| 45-54 (vs. 22-34) | 0.925 | 0.626 | 1.367 |
| 55-64 (vs. 22-34) | 1.501 | 0.942 | 2.393 |
| 65+ (vs. 22-34) | 1.432 | 0.915 | 2.242 |
| Mental distress | 0.781 | 0.567 | 1.075 |
| Self-reported poor health status | 1.088 | 0.752 | 1.574 |
| Chronically ill | 1.392 | 0.985 | 1.966 |
| Population group |  |  |  |
| Ultra-Orthodox (vs. non-Orthodox Jews) | 1.465 | 0.89 | 2.411 |
| Arabs (vs. non-Orthodox Jews)*** | 0.17 | 0.122 | 0.235 |
| Residence in peripheral areas | 1.676 | 1.144 | 2.456 |
| HP |  |  |  |
| Macabi (vs. Clalit) | 0.678 | 0.48 | 0.958 |
| Meuhedet (vs. Clalit)*** | 0.461 | 0.311 | 0.683 |
| Leumit (vs. Clalit)*** | 0.455 | 0.28 | 0.742 |
| Nonacademic education | 0.816 | 0.61 | 1.092 |
| Income quintile*** |  |  |  |
| Higher (vs. lowest)*** | 6.56 | 3.041 | 14.147 |
| 2nd (vs. lowest)*** | 6.282 | 3.437 | 11.481 |
| 3rd (vs. lowest)*** | 4.987 | 2.957 | 8.41 |
| 4th (vs. lowest)*** | 2.307 | 1.701 | 3.129 |
| Constant | 2.64 |  |  |
| Hosmer and Lemeshow Test | Chi-square | df | Sig. |
|  | 15.597 | 8 | 0.049 |

*p < 0.05; **p < 0.01; ***p < 0.001

1. The potential accessibility component expresses the proximity of the locality to each of the localities, and isweighted by the size of their population.The size of the population indicates the intensity of the possibilities, activities and assets in the locality. The proximity component to the Tel Aviv district border expresses the monocentric structure of the State of Israel, in which the Tel Aviv districtis the economic and business center of the state.The low value of the index indicates the most peripheral locality, and the high value – the most central locality<https://tinyurl.com/27xpuvk2>))).

   [↑](#footnote-ref-1)
